# Main barriers on glucometer utilization during physician's appointment of insulin users T2D patients

**DOI:** 10.1186/1758-5996-7-S1-A201

**Published:** 2015-11-11

**Authors:** Cristal Peters Cabral, Erika Bezerra Parente, Paula Vieira Freire, Andre Carvalho Yamaya, Caroline Schnoll, Vivian Roberta Ferreira Simões, Alessandra Muto, João Eduardo Nunes Salles

**Affiliations:** 1 Santa Casa de Misericordia de São Paulo, São Paulo, Brazil

## Background

Self-monitoring blood glucose (SMBG) is an important tool for type 2 diabetes treatment, especially for insulin users. However, several patients that receive this device from government do not make the proper use of it.

## Objective

Investigate the main barriers for glucometer utilization to evaluate glycemic control of insulin users T2D patients, during physician's appointment.

## Method

Glycemic data was obtained from patient's glucometers by using Accu Chek 360 software for downloading. We used data of all insulin users T2D patients that came for physician's appointment at the diabetes unit in a public hospital, in the city of Sao Paulo, from March to June 2015. A survey regarding the glucometer usage was applied.

## Results

**Figure 1:**
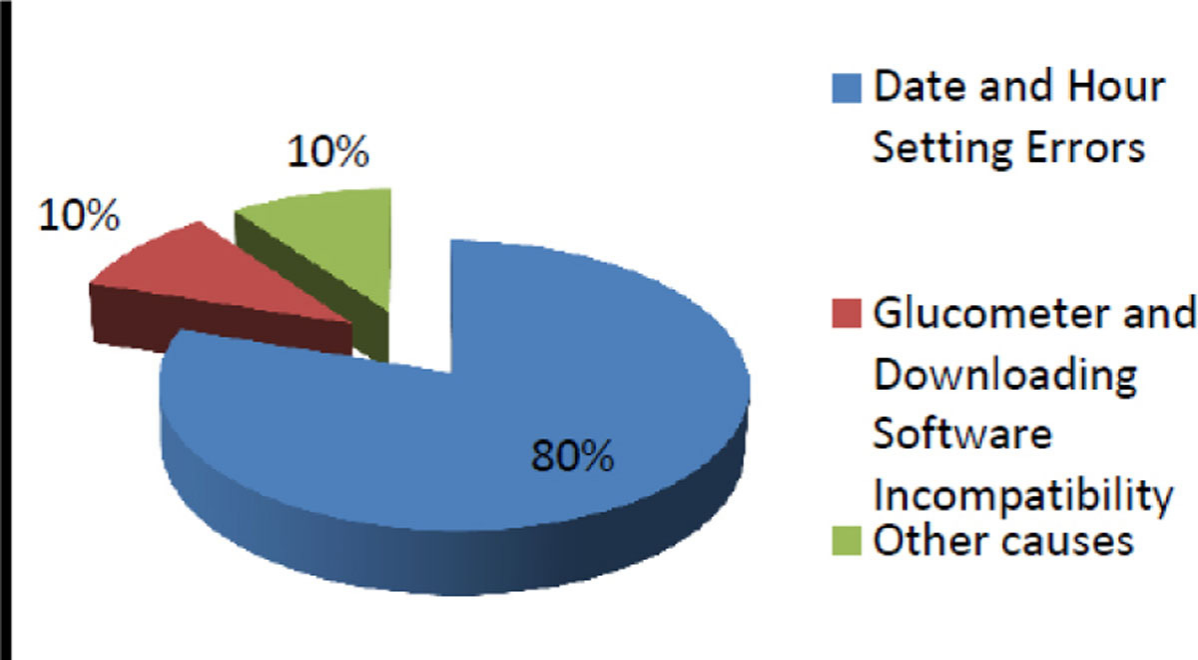
Readons that it was not possible to use glucometer information.

**Figure 2:**
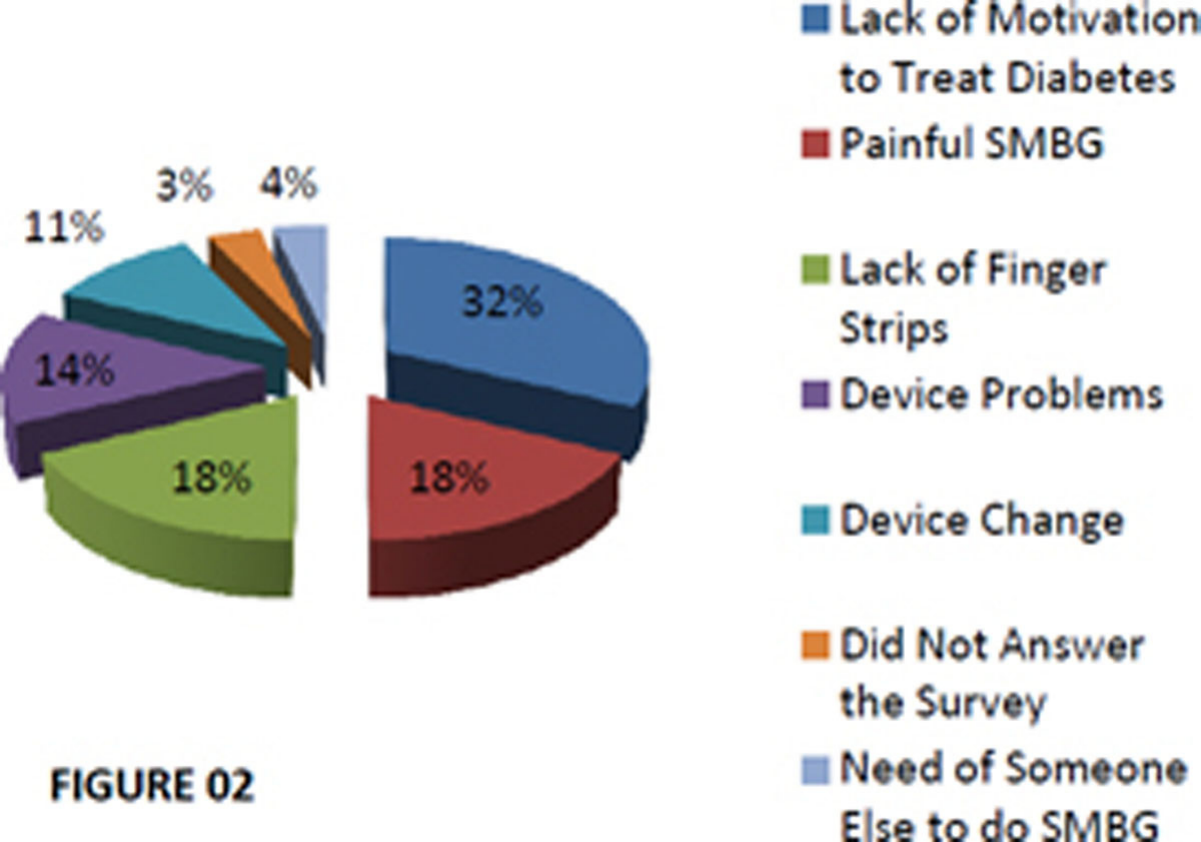
Reasons that less than 90 blood glucose tests were performed.

From a total of 417 patients, 95 were eligible to this analisys. It was not possible to use glucometer information in 31.6% of 95 patients because the reasons on figure 1. It was suitable to collect data from 68.4% of 95 patients, nevertheless, 43.1% of these performed less than 90 blood glucose tests (less than once a day) and causes are shown in figure 2.

## Conclusion

Insulin users T2D patients receive glucometer and supplies from government for free, however the device information was useful only in 56.9% of all cases. The main reason was lack of information how to use the device and the second was lack of motivation to keep diabetes treatment. Our results showed that we need more education programs for our patients becouse giving glucometer without education will not help diabetes treatment.

